# Taxonomic study of the genus *Diolcogaster* Ashmead (Hymenoptera, Braconidae, Microgastrinae) from Borneo with the description of four new species

**DOI:** 10.1371/journal.pone.0332071

**Published:** 2025-10-15

**Authors:** Geng Lu, Shiqi Chen, Yaoyue Lei, Mengqi Wen, Zhen Liu, Mostafa Ghafouri-Moghaddam, Andrew Polaszek

**Affiliations:** 1 Agricultural Products Processing and Food Safety Key Laboratory of Hunan Higher Education, Hunan University of Arts and Science, Changde, China; 2 Zoology Key Laboratory of Hunan Higher Education, College of life and environmental sciences, Hunan University of Arts and Science, Changde, China; 3 Integrative Insect Ecology Research Unit, Department of Biology, Faculty of Science, Chulalongkorn University, Bangkok, Thailand; 4 Science: Research, Natural History Museum, London, United Kingdom; Federal University of Espirito Santo: Universidade Federal do Espirito Santo, BRAZIL

## Abstract

Taxonomic studies of Microgastrinae (Hymenoptera: Braconidae) are seldom conducted on material from Borneo, despite its being widely recognized as one of the world’s biodiversity hotspots. As part of our preliminary studies on the braconids from this island in the Natural History Museum (UK) collection, we describe and illustrate four new species of *Diolcogaster* Ashmead: *D. dolichogaster* Liu & Polaszek, **sp. nov.**, *D. flavicoxa* Liu & Polaszek, **sp. nov.**, *D. hamus* Liu & Polaszek, **sp. nov.**, and *D. parallela* Liu & Polaszek, **sp. nov.** Two previously described species, *D. eclectes* (Nixon) and *D. urios* (Nixon), are redescribed and illustrated. The species examined in this study are classified into two distinct groups, the *basimacula*–group (*D. dolichogaster, D. eclectes,* and *D. hamus*) and the *xanthaspis*–group (*D. flavicoxa, D. parallela and D. urios*). Additionally, we provide an identification key to the six *Diolcogaster* species from Borneo, with a distribution map. A concise discussion of the *Diolcogaster* species–groups is provided.

## Introduction

The subfamily Microgastrinae is the most diverse subfamily of Braconidae and represents a vast assemblage of species worldwide, estimated to number between 17,000 and 46,000 [1], of which 2,999 have been described [2]. Microgastrinae are all koinobiont endoparasitoids on lepidopteran larvae, with many species utilised as biological control agents against pest species [2–4]. Microgastrines are particularly diverse in the Oriental region, especially in Southeast Asia; however, the fauna remains poorly studied.

*Diolcogaster* Ashmead, 1900, is a cosmopolitan genus with 143 described species known from all biogeographical regions of the world [2,5]. The estimated number of undescribed *Diolcogaster* species ranges from 100 to 1,000 globally, especially in tropical regions, based on available museum specimens and DNA barcoding data (e.g., [2]). There are 6,233 DNA barcode sequences of this genus in BOLD (Barcode of Life Database, http://www.boldsystems.org/, 25th June, 2025), representing 432 BINs, most of them from Costa Rica and Canada. Only one sequence among them (CNCH0894) was recorded from Borneo based on the decimal degrees (3.671661N, 114.6333E), placing it in Sarawak, just south of Brunei and fairly close to Gunung Mulu NP where several specimens in the current study were collected. As for the surrounding countries, 24 sequences representing 13 BINs from Malaysia, 55 sequences representing 12 BINs from Indonesia, and 14 sequences representing 5 BINs from Papua New Guinea are recorded, no records concern the parts of those countries located in Borneo (Kalimantan-Indonesia; Sabah and Sarawak-Malaysia). Consequently, the current number of described species remains extremely low and is limited to a few studies [e.g., 6–13]. Most species, particularly the older ones, have never been collectively examined, and existing information is outdated, with poor descriptions, minimal illustrations, no identification keys, and questionable species–group assignments.

Despite being one of the dominant genera within Microgastrinae, *Diolcogaster* remains poorly understood, particularly in the highly diverse tropical regions. Historically, the genus was treated under the names *Microgaster* Latreille (e.g., [14]) and *Protomicroplitis* Ashmead [6]. Nixon [6] placed 44 *Diolcogaster* species in *Protomicroplitis*, which he divided into 22 species–groups, describing several new taxa, mainly from the Neotropics and Southeast Asia. However, his classification, including *Protomicroplitis*, remained incomplete, as many of the newly proposed genera and species–groups were based on limited information (e.g., species or genera described from a single specimen, often relying solely on body colour), particularly for taxa distributed in the Oriental region. In his benchmark study on Microgastrinae classification, Mason [3] later reconstructed the generic classification of Microgastrinae based on an intuitive phylogenetic framework. Mason [3] redefined *Protomicroplitis* in a much narrower sense, reinstated *Diolcogaster* as a valid genus, and transferred most species previously assigned to *Protomicroplitis* (*sensu* [6]) to *Diolcogaster*, providing a more accurate and detailed description based on the type species, *Microgaster brevicaudus* Provancher. He incorporated 17 of Nixon’s *Protomicroplitis* species–groups, comprising 24 species, into *Diolcogaster*. Tobias [15] listed 10 *Diolcogaster* species, including five new combinations, in his faunal study of the European part of the USSR. Saeed et al. [9] investigated the diversity and evolutionary significance of *Diolcogaster* and revised 26 Australasian species. Fernández-Triana [11] revised the status of several *Protomicroplitis* species and transferred 13 of them to *Diolcogaster*.

*Diolcogaster* is relatively easy to distinguish from other microgastrine genera, even though it is rather diverse morphologically [16]. *Diolcogaster* presents several unresolved phylogenetic issues, and its monophyly has been questioned by many systematists (e.g., [9]). To date, the genus has rarely been investigated in a rigorous phylogenetic framework, though it is postulated to be polyphyletic by Fernández-Triana et al. [2]*.* Additionally, many of its recognized species–groups are highly distinctive, with some appearing more closely related to other genera (e.g., [9,16]). As a result, developing a phylogenetic classification for *Diolcogaster* is integral to resolving broader taxonomic uncertainties within Microgastrinae, particularly within the large and complex *Cotesia* group (*sensu* [3]).

*Diolcogaster* species are well-suited to parasitize exposed Lepidoptera larvae, such as those of many macrolepidoptera [2]. Thirteen Lepidoptera families were listed as the hosts of *Diolcogaster* by Seed et al. [9], many of which are significant agricultural and forestry pests. A few species, such as *D. facetosa*, the most common North American species, have been employed as biological control agents [17]. However, based on their biology, many other *Diolcogaster* species may also have potential for pest management.

Although some taxonomic revisions have been conducted in different biogeographic regions (see [2]), only two species have been reported from Borneo (Malaysia, Sabah): *D. eclectes* (Nixon, 1965) and *D. urios* (Nixon, 1965). No additional records or revisions of this genus exist for the island. Borneo remains largely unexplored in terms of Microgastrinae diversity, despite being one of the world’s biodiversity hotspots. As part of our preliminary studies on the braconids of this island, we examined specimens from the Natural History Museum, London, and discovered four new *Diolcogaster* species from Borneo, which we describe and illustrate here. Additionally, we redescribe and illustrate the two previously described *Diolcogaster* species from the island, provide a distribution map, and present an identification key for all known species of the genus from Borneo.

## Materials and methods

Descriptions and measurements were conducted using a Zeiss Stemi SV6 stereomicroscope. Photographs were captured with a digital camera (Zeiss AxioZoom or Hirox HRX-01) and processed using Helicon Focus software. Further image enhancements were done in Adobe Photoshop CS6. Morphological terminology for body structures and measurements primarily follows Gupta and Fernandez-Triana [12] and Zeng et al. [10]. Wing vein terminology follows the modified Comstock–Needham system [18], while cuticular sculpture terminology follows Harris [19]. The following abbreviations are used in this study: **POL **= postocellar line; **OOL **= ocular-ocellar line; **OD **= ocellar diameter; **T1 **= first metasomal tergite; **T2 **= second metasomal tergite; **T3 **= third metasomal tergite. The new species described in this study are deposited in the Natural History Museum, UK (**NHMUK**), while the examined holotypes of two previously described species are housed in the United States National Museum, Washington, D.C., USA (**USNM**).

### Nomenclatural acts

The electronic edition of this article conforms to the requirements of the amended International Code of Zoological Nomenclature, and hence the new names contained herein are available under that Code from the electronic edition of this article. This published work and the nomenclatural acts it contains have been registered in ZooBank, the online registration system for the ICZN. The ZooBank LSIDs (Life Science Identifiers) can be resolved and the associated information viewed through any standard web browser by appending the LSID to the prefix ““http://zoobank.org/”“. The LSID for this publication is: urn:lsid:zoobank.org:pub: E7B4A976-F0C0-4D7C-8403-A966FC387862. The electronic edition of this work was published in a journal with an ISSN, and has been archived and is available from the following digital repositories: PubMed Central, LOCKSS.

## Results

### Key to the *Diolcogaster* species from Borneo

**Fig 1:**
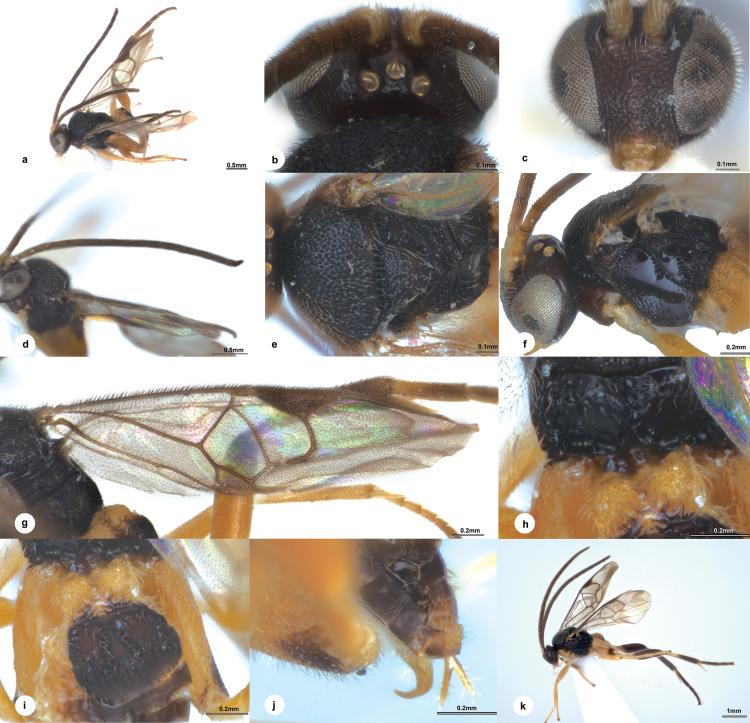
*Diolcogaster flavicoxa* Liu & Polaszek, sp. nov., holotype female (NHMUK). **a–j** holotype female, **k** paratype male. **a** habitus, lateral view **b** head, dorsal view **c** head, frontal view **d** antenna **e** mesosoma, dorsal view **f** mesosoma, lateral view **g** fore wing **h** propodeum **i** T1–T3 **j** ovipositor sheaths **k** paratype male, lateral view.

**Fig 2:**
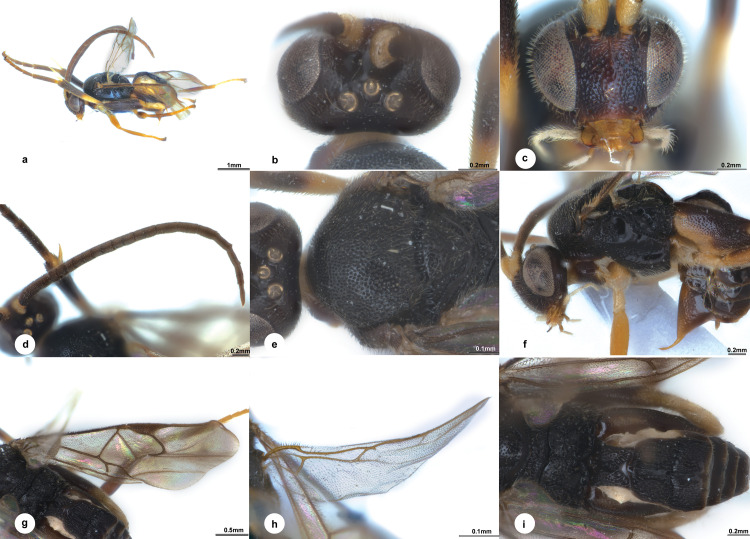
*Diolcogaster parallela* Liu & Polaszek, sp. nov., holotype female (NHMUK). **a** habitus, lateral view **b** head, dorsal view **c** head, frontal view **d** antenna **e** mesonotum **f** mesosoma and metasoma, lateral view **g** fore wing **h** hind wing **i** propodeum and metasoma, dorsal view.

**Fig 3:**
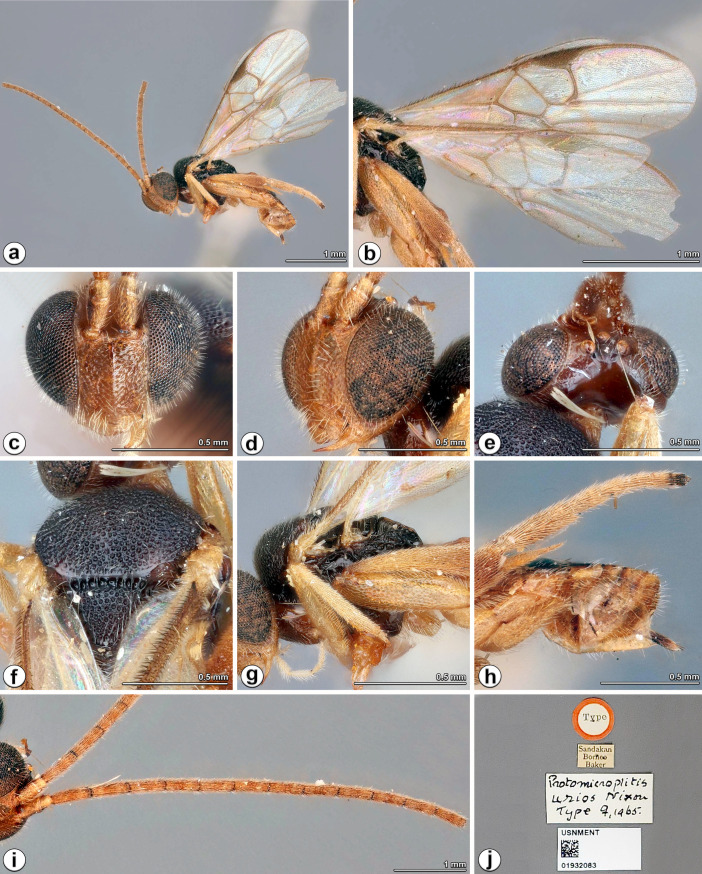
*Diolcogaster urios* (Nixon, 1965), holotype female (USNM). **a** habitus, lateral view **b** wings **c** head, frontal view **d** head, frontolateral view **e** head, dorsal view **f** mesoscutum, dorsal view, **g** mesosoma, lateral view **h** metasoma, lateral view **i** antenna **j** type labels. Source: from ©USNM, reproduced under a CC BY license, with permission from USNM [2025].

**Fig 4:**
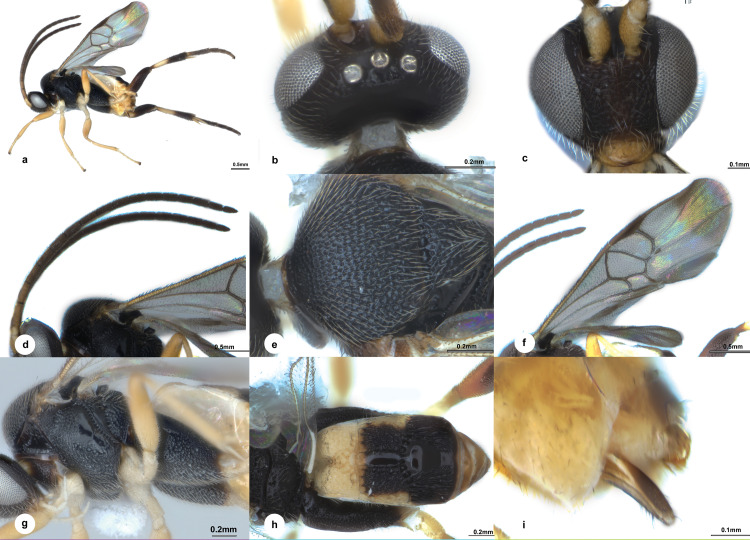
*Diolcogaster dolichogaster* Liu & Polaszek, sp. nov., holotype female (NHMUK). **a** habitus, lateral view **b** head, dorsal view **c** head, frontal view **d** antenna **e** mesonotum **f** fore wing **g** mesosoma and metacoxa, lateral view **h** propodeum and metasoma, dorsal view **i** ovipositor sheaths.

**Fig 5:**
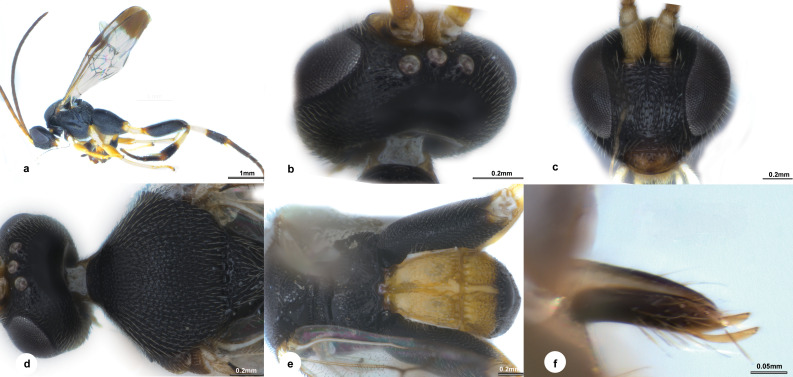
*Diolcogaster eclectes* (Nixon, 1965), non-type female (NHMUK). **a** habitus, lateral view **b** head, dorsal view **c** head, frontal view **d** mesonotum, dorsal view **e** propodeum and metasoma, dorsal view **f** ovipositor sheaths, lateral view.

**Fig 6:**
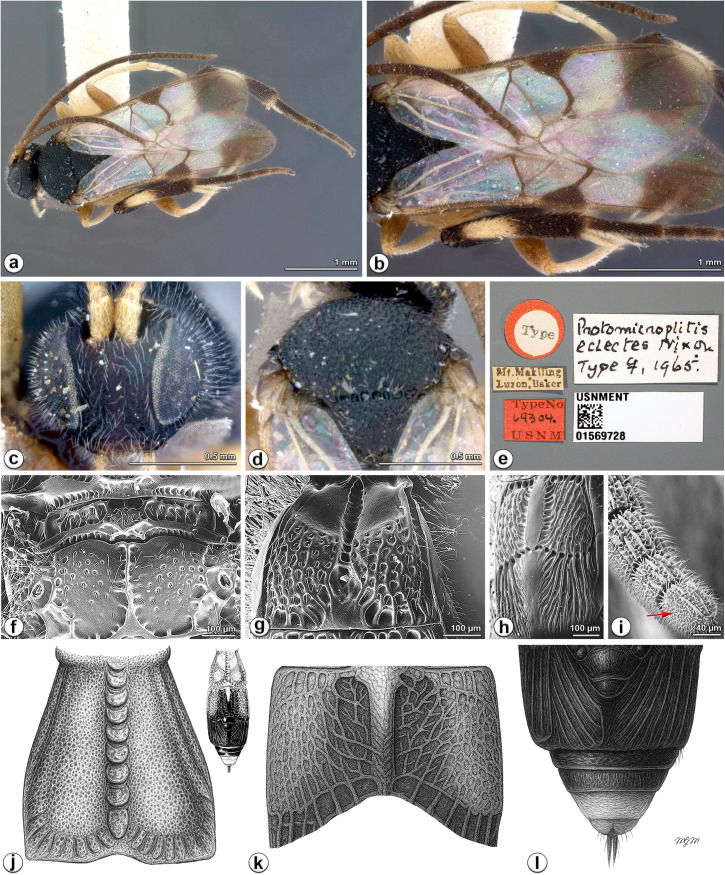
*Diolcogaster eclectes* (Nixon, 1965), holotype female (USNM). **a** habitus, dorsal view **b** wings **c** head, frontal view **d** mesoscutum, dorsal view **e** type labels **f** propodeum, dorsal view **g** T1, dorsal view **h** T2–T3, dorsal view **i** ventral surface of apical antennal flagellomeres lacking placodes (red arrow) **j** T1, dorsal view **k** T2, dorsal view **l** T3–T7, dorsal view. Source for figs a–e: from ©USNM, reproduced under a CC BY license, with permission from USNM [2025]. The original images in panels f–i are from Saeed et al. (1999) and have been modified and reproduced here. The original illustration, shown as a small image in the top right of Fig 5j, was sourced from Kotenko (2007) and subsequently redrawn in Figs. j–l.

**Fig 7:**
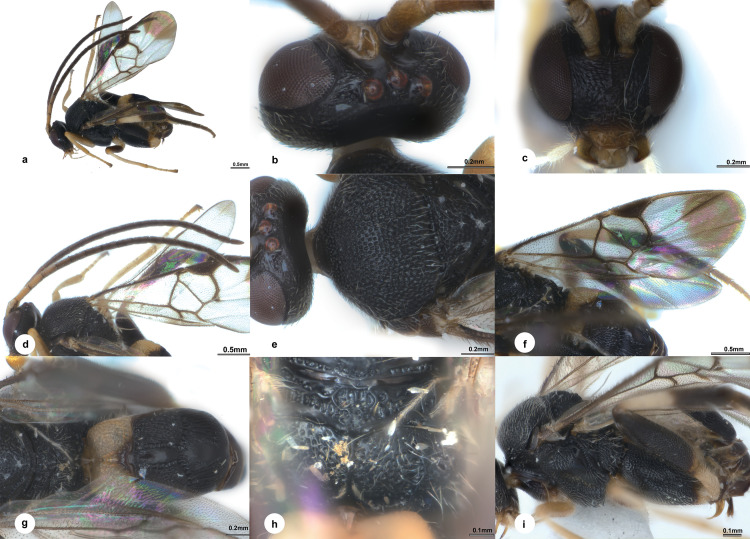
*Diolcogaster hamus* Liu & Polaszek, sp. nov., holotype female (NHMUK). **a** habitus, lateral view **b** head, dorsal view **c** head, frontal view **d** antenna **e** mesonotum **f** fore wing **g** metasoma, dorsal view **h** propodeum **i** mesosoma and metasoma, lateral view.

1– Fore wing without distinct dark apical patch (Figs 1g, 2g, 3b); T1 narrowed, constricted to apex (Figs 1i, 2i).... **2** [*xanthaspis*–group]
**–** Fore wing with distinct dark apical patch (Figs 4f, 5a, 6b, 7f); T1 broad, widening to apex (Figs 4h, 5e, 7g).... **4** [*basimacula*–group]
2– Head red-yellow (Fig 3e); vein r-m of fore wing connected to meeting point of r and 2-SR (Fig 3b); areolet large, triangular (Fig 3b).... ***D. urios* (Nixon)**
**–** Head dark brown to black (Figs 1b, 2b); vein r-m of fore wing connected to 2-SR (Figs 1g, 2g); areolet small, slit-like (Figs 1g, 2g).... **3**
3– Metacoxa, T1 and metafemur largely yellow (Fig 1i); punctures more rugulose and denser on face (Fig 1b); median field of T2 widened towards apex (Fig 1i); T3 with sparse obsolete weak striation (Fig 1i).... ***D. flavicoxa* Liu & Polaszek, sp. nov.**
**–** Metacoxa, T1 and metafemur largely black or dark brown (Fig 2i); punctures slightly rugulose and discrete on face (Fig 2b); median field of T2 parallel-sided (Fig 2i); T3 strongly and densely striate (Fig 2i).... ***D. parallela* Liu & Polaszek, sp. nov.**
4– Ovipositor sheath with obvious spatulate, upcurved strong setae (Fig 5f); T2 yellow in larger area (Fig 5e); median field of T2 narrower and without strong striation next to it (Figs 5e, 6k).... ***D. eclectes* (Nixon)**
**–** Ovipositor sheath without spatulate, upcurved setae (Figs 4i, 7); T2 black, only sometimes with limited yellow part laterally (Figs 4h, 7g); median field of T2 broader and with strong striation next to it (Figs 4h, 7g).... **5**
5– Propodeum largely polished (Fig 4h); vertex with few sparse punctures between eye and hind ocellus (Fig 4b); vein 1-SR indistinct (Fig 4f)....***D. dolichogaster* Liu & Polaszek, sp. nov.**
**–** Propodeum entirely coarsely densely punctate (Fig 7h); vertex with transverse rugulose punctures between eye and hind ocellus (Fig 7b); vein 1-SR much longer (Fig 7f)....***D. hamus* Liu & Polaszek, sp. nov.**

## Taxonomy

### *Diolcogaster dolichogaster* Liu & Polaszek, sp. nov.

urn:lsid:zoobank.org:act:E4E5FCC5–0327-485E-A4DB-C3924B876F78

**Material examined** (NHMUK)**. HOLOTYPE**: 1♀, MALAYSIA, Borneo, Sabah, DMQI Sept/Oct 2012, Maliau Plot 7, Trap MT2, No. NHMUK010826500. **PARATYPES**: 1♀, MALAYSIA, Borneo, Sarawak, 4^th^ Div. G[u]n[ung]. Mulu RGS Exp. (Malaise trap), NM Collins, v.1978, No. NHMUK010826499; 1♀, MALAYSIA, Pahang, F[ederated].M[alay].S[tates]. Fraser’s Hill, 4200 ft,18.vi.1951 Ex F.M.S. Museum.B.M.1955−354. No. NHMUK010826498.

**Diagnosis.** Body 3.2 mm long, black, except T1, lateral parts of T2 and tergites posterior to T3 yellow to light yellow brown (Fig 4a); head 1.9 × as wide as long; eyes 1.7 × longer than temple in dorsal view; POL:OD:OOL = 2.2:1.0:1.1; vertex with indistinct punctures (Fig 4b); face slightly wider than high (Fig 4c); antenna (Fig 4d) with penultimate flagellomere 2.1 × longer than wide; puncture intervals on mesoscutum with micro punctuation (Fig 4e); propodeum largely highly polished on dorsal part (Fig 4h); mesopleuron areolate-rugose on lower half and anterior part (Fig 4g); pterostigma 4.2 × as long as its widest part; vein r 1.3 × 2-SR; areolet medium-sized, 3-sided; vein 1-CU1 0.8 × 2-CU1 (Fig 4f); metacoxa relatively shorter, reaching posterior margin of T2, evenly covered with dense umbilical punctures (Fig 4g); T1 gradually widened towards apex, 1.5 × longer than basal width, radiate carinae weak, elsewhere shallowly punctate entirely (Fig 4h); T2 with a median field parallel-sided, elsewhere with strong striations and ill-defined punctures in between, 1.8 × as long as midlength; T3 1.2 × as long as T2, with median polished field broad at base and gradually narrowed toward apex, sparsely striate laterally; ovipositor without modified setae at apex (Fig 4i).

**Description.** Female. Body length 3.2 mm, fore wing length 3.3 mm (Fig 4a).

***Head.*** 1.9 × as wide as long, as wide as mesoscutum. Eyes 1.7 × longer than temple in dorsal view (Fig 4b). Temple a little shiny with shallow punctures, constricted behind eyes in dorsal view. Vertex shiny with small indistinct punctures. Ocelli small, distance between fore and a hind ocellus 0.6 × as long as minor axis of an hind ocellus, POL:OD:OOL = 2.2:1.0:1.1. Frons shiny and polished. Face less shiny with setigerous-punctate, weakly rugulose below sockets, a carina present at upper half midlongitudally, slightly wider than high. Clypeus 2.5 × wider than medial length, nearly smooth entirely. Tentorial pits of moderate size, distance between tentorial pits 3.0 × as long as distance from pit to eye margin. Length of malar space 1.3 × width of mandible (Fig 4c). Antenna 1.1 × longer than body length, with 1st, 2nd and penultimate flagellomeres 3.1, 2.4 and 2.1 × longer than wide, flagellomeres gradually shortened to penultimate flagellomere, closely articulated (Fig 4d).

***Mesosoma*.** Length:width:height = 1.5:1.0:1.3. Mesoscutum a little shiny with dense punctures, its intervals less than one puncture diameter and with micro wrinkles, narrowly polished along hind margin; notauli not impressed. Scutellar sulcus wide and straight with 10 carinae inside (Fig 4e). Disc of scutellum a littly shiny with similar punctures as mesoscutum, polished at tip, so the posterior, polished band of scutellum is continuous; lateral part of polished band of scutellum reaching over half of disc of scutellum with the anterior triangular part narrowly depressed with shallow sparse punctures. Propodeum 3.2 × wider than high, shiny with strong percurrent midlongitudinal carina, largely highly polished on dorsal part, spiracles large, enclosed by costulae (Fig 4h). Mesopleuron shiny, areolate-rugose on lower half and anterior part, only narrowly polished on medio-posterior part and few discrete punctures underneath, precoxal sulcus indistinct covered by rugosity (Fig 4g).

***Wings.*** Fore wing (Fig 4f): pterostigma narrow, 4.2 × as long as its widest part; vein 1-R1 1.5 × length of pterostigma; vein r slightly obliquely arising from apical 2/5 of pterostigma, 1.9 × longer than maximum width of pterostigma, meeting vein 2-SR at a 138 degree angle, 1.3 × 2-SR; vein r-m 0.4 × 2-SR; areolet medium, 3-sided; vein m-cu 1.5 × 2-SR + M; vein 1-CU1 0.8 × 2-CU1 and 1.1 × cu-a. Hind wing: narrow, with edge of vannal lobe beyond its widest part nearly straight and with minute setae; cu-a straight.

***Legs.*** Metacoxa relatively shorter, reaching posterior margin of T2, flattened on outer side, evenly covered with dense umbilical punctures, the interspaces shiny (Fig 4g). Metafemur 3.3 × as long as its widest part. Metatibia gradually swollen toward apex and 0.7 × as long as metatarsus. Inner metatibial spur much longer than outer one, 0.7 × as long as metabasitarsus; fourth tarsal segment as long as fifth tarsal segment; apical segment of the front tarsus without a spine (Fig 4a). Tarsal claws simple.

***Metasoma*.** 1.5 × longer than mesosoma. T1 mediumly incurved from lateral view, gradually widened towards apex, 1.5 × longer than basal width, with complete longitudinal groove attaching to weak short radiate carinae arising from apical knob of T1, elsewhere shallowly punctate entirely (Fig 4h). T2 nearly rectangular, with a median field parallel-sided and encircled by crenulate grooves, elsewhere with strong striations and ill-defined punctures in between, 1.8 × as long as midlength. T3 transverse, 1.2 × as long as T2, with median polished field broad at base and gradually narrowed toward apex, sparsely striate laterally, membranous and narrowly polished on posterior margin. Tergites posterior to T3 contracted, membranous. Setae of metasoma very sparse. Ovipositor sheath narrow, relatively densely setose on apical third, without modified setae at apex; ovipositor thin and slightly curved (Fig 4i). Hypopygium large, evenly sclerotised, smooth with small punctures, not surpassing the last tergite.

***Colour.*** Body black, except T1, lateral parts of T2 and tergites posterior to T3 yellow to light yellow-brown (Fig 4a). Palpi white. Tibia spurs pale yellow. Antenna brown to dark brown except scape and pedicel pale yellow-brown. Legs pale yellow to yellow, except metacoxa black, most of metafemur, apical half of metatibia and metatarsus dark brown. Wing membranes hyaline, fore wing marked with brown patches at apex, pterostigma brown, veins pale brown to brown, r-m hyaline.

**Variation.** No distinct differences observed among specimens. Body length 3.2–3.3 mm, fore wing length 3.3–3.4 mm.

**Male.** Unknown.

**Host.** Unknown.

**Fig 8:**
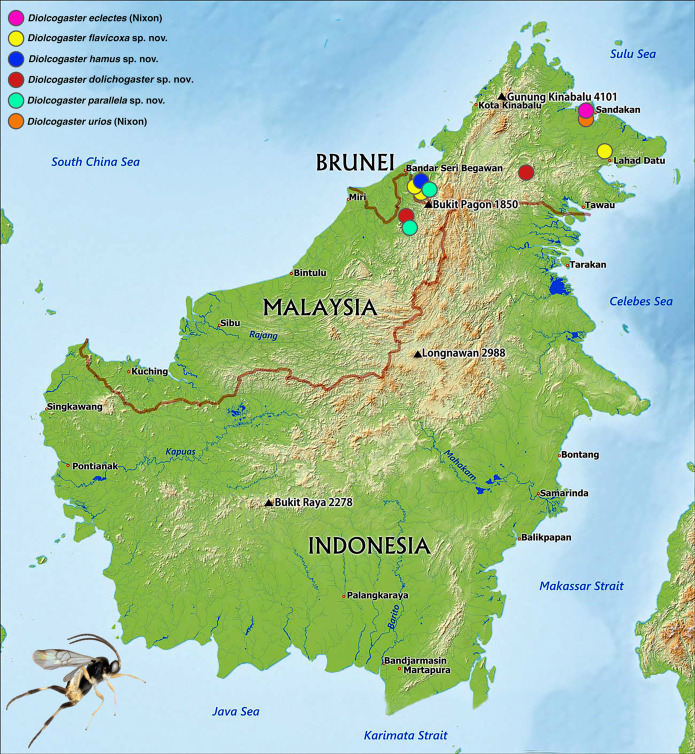
Distribution map of all known *Diolcogaster* species recorded from Borneo. Source: from ©Blue Green Atlas, reproduced under a CC BY license, with permission from Blue Green Atlas [2025].

**Distribution** (Fig 8)**. Oriental** (Borneo [Malaysia-Sarawak and Sabah]; Malaysia-Pahang).

**Etymology.** The specific name “*dolichogaster*” derives from the Greek “dolichos-” (=long), and “gaster”, referring to its long metasoma.

**Remarks.** This species is similar to *D. eclectes*, but differs in the following: propodeum largely polished dorsally (densely punctate in *D. eclectes*); metacoxa with dense umbilical punctures laterally (with sparse minute punctures in *D. eclectes*); and T2 with strong striations laterally (without striations in *D. eclectes*). *Diolcogaster dolichogaster* belongs to the *basimacula*–group which can be distinguished from other species–groups by the following combination of characters: i) fore wing areolet slit-like; ii) hind wing vannal lobe concave and glabrous; iii) metasomal tergites form a partial carapace; iv) T2 and T3 with well-defined median field; v) suture between T2 and T3 deep, wide, and crenulate; vi) metacoxa approximately 1.6 × as long as T1 or longer; vii) ventral area of the lateral pronotum crenulate, with both dorsal and ventral grooves; viii) propleural flange not well developed [6,9]. We examined the images of the various BIN specimens deposited in BOLD from the surrounding countries, none of which were a match.

### *Diolcogaster eclectes* (Nixon).

*Protomicroplitis eclectes*: Nixon 1965: 246 [6]; Rao & Chalikwar 1970: 112–113 [20].

*Protomicroplitis eclectes extentus*: Papp 1974: 165–175 [21].

*Diolcogaster eclectes*: Mason 1981: 115 [3]; Kotenko 2007: 162–163 [22]; Fernández-Triana et al. 2020: 399–400 [2].

**Material examined. HOLOTYPE**: female, **PHILIPPINES**, Laguna Province, Luzon, Mount Makiling, CF Baker coll., type No. 9304, USNMENT01569728 (images examined).

**Additional material** (USNM)**. PARATYPES**: 1♂, same data as holotype; 1♂, PHILIPPINES, Mindanao, Davao, CF Baker coll.; 1♂, MALAYSIA, Penang, CF Baker coll.; 1♂, SINGAPORE, CF Baker coll.; 1♂, MALAYSIA, Borneo, Sabah, Sandakan, CF Baker coll.; **NONTYPES** (NHMUK): 1♀, MALAYSIA, Borneo, Sabah, Danum-Maliau Quantitative Inventory (DMQI) Sept/Oct 2012, Maliau Plot -, Trap MT1, No. NHMUK010826502; 1♀, same data except Danum Plot 1, Trap MT2, No. NHMUK010826501; 1♂, same data except Maliau Plot 3, Trap MT2, No. NHMUK 010826503.

**Diagnosis.** Fore wing with distinct dark apical patch, vein r of fore wing and 2–SR forming an obtuse angle, 2^nd^ discal cell almost always setose (Figs 5a, 6b); edge of vannal lobe beyond its widest part, very slightly concave, without fringe of setae; flagellomeres more slender in apical half and smooth; propodeum smooth with a median longitudinal carina, its sculpture reduced to coarse, obsolescent punctation (Figs 5e, 6f); T1 broad, widening to apex with a distinct median longitudinal groove (Figs 5e, 6g); T2 yellow in larger area (Fig 5e); median field of T2 narrower and without strong striation next to it (Figs 5e, 6k); T3 heavily sclerotised as T2, forming a sculptured carapace with median subtriangular and polished area and striae on sides, widened in anterior; tergite less narrowed to apex, yellow throughout; ovipositor sheath with obvious spatulate, upcurved strong setae (Fig 5f); metacoxa almost smooth with sharp and mostly discrete punctures of various sizes; metafemur red-brown; metatibia yellow on about basal half and dark at extreme base (Fig 5a).

**Redescription.** Female. Body length 3.1–4.1 mm, fore wing length 3.6 mm (Fig 5a).

***Head.*** 2.2 × as wide as long, 0.9 × as wide as mesoscutum. Eyes 1.4 × longer than temple in dorsal view (Fig 5b). Temple shiny with shallow punctures, slightly constricted behind eyes in dorsal view. Vertex shiny with weak transverse wrinkles between ocelli and eye, largely polished behind ocelli. Ocelli small, distance between fore and a hind ocellus 0.4 × as long as minor axis of an hind ocellus, POL:OD:OOL = 1.8:1.0:1.7 (Fig 5b). Frons shiny and polished. Face a little shiny with strong minute punctures, 0.9 × as wide as high. Clypeus 3.6 × wider than medial length, rugulose entirely. Tentorial pits of moderate size, distance between tentorial pits 3.4 × as long as distance from pit to eye margin. Length of malar space 1.5 × width of mandible (Figs 5b, 6c). Antenna 1.1 × longer than body length, with 1st, 2nd and penultimate flagellomeres 3.5, 3.0 and 1.9 × longer than wide, flagellomeres gradually shortened to penultimate flagellomere, closely articulated (Figs 5a, 6a).

***Mesosoma*.** Length:width:height = 2:1.0:1.1. Mesoscutum a little shiny with dense punctures, its intervals shorter than one puncture diameter with micro wrinkles, except nearly polished along hind margin; notauli not impressed (Figs 5d, 6d). Scutellar sulcus wide and straight with 8 carinae inside. Disc of scutellum slightly shiny with small dense punctures entirely, polished at tip, so the posterior, polished band of scutellum is continuous; lateral part of polished band of scutellum reaching hardly to half of disc of scutellum with the anterior triangular part nearly smooth. Propodeum 2.4 × wider than high, shiny with strong percurrent midlongitudinal carina, smooth with its sculpture reduced to coarse, obsolescent punctation (Figs 5e, 6f). Mesopleuron shiny, largely smooth except dense rugose punctures anteriorly, precoxal sulcus depressed with sparser punctures, setigerous-punctate ventrally (Fig 5a).

***Wings.*** Fore wing (Figs 5a, 6b): pterostigma large, 2.2 × as long as its widest part; vein 1-R1 1.3 × length of pterostigma; vein r obliquely arising from apical 2/5 of pterostigma, indistinctly longer than maximum width of pterostigma, meeting vein 2-SR at a 133 degree angle, 1.4 × longer than 2-SR, the latter distinctly segmented by r-m; areolet obvious, 3-sided; vein m-cu about as long as 2-SR + M; vein 1-CU1 0.8 × 2-CU1 and 1.3 × cu-a. Hind wing: narrow, with edge of vannal lobe beyond its widest part very slightly concave and without trace of a fringe of setae, cu-a nearly straight.

***Legs.*** Metacoxa very large, reaching beyond posterior margin of T3, flattened on outer side, evenly densely covered with fine and sparse punctures, the interspaces smooth and shiny (Fig 5a). Metafemur 3.4 × as long as its widest part. Metatibia swollen toward apex and 0.8 × as long as metatarsus. Inner metatibial spur much longer than outer one, about 0.8 × as long as metabasitarsus; fourth tarsal segment indistinctly shorter than fifth tarsal segment; apical segment of the front tarsus without a spine (Figs 5a, 6a). Tarsal claws simple.

***Metasoma*.** 1.1 × longer than mesosoma. T1 vertically incurved from lateral view, broad, widening to apex, 1.9 × longer than basal width, about as long as apical width, with a complete median longitudinal groove, elsewhere shallowly punctate entirely (Figs 5e, 6g, 6j); T2 trapezoid, with a narrow, parallel-sided median field and without strong striation next to it except rugulose punctures, 1.4 × as long as midlength (Fig 6k). T3 transverse, 0.8 × as long as T2, heavily sclerotised as T2, forming a sculptured carapace with median subtriangular and polished area and striae on sides, widened in anterior, separated from T2 by a crenulate groove (Fig 5e). Tergites posterior to T3 more membranous. Setae of metasoma very sparse. Ovipositor sheath relatively densely setose, with obvious spatulate, upcurved strong setae (Fig 5f). Hypopygium medium-sized, evenly sclerotised, smooth with sparse fine setae, not surpassing the last tergite.

***Colour.*** Body black, slightly brown, except T1 and most of T2 yellow to pale yellow (Figs 5a, 6a). Palpi white. Tibia spurs pale yellow. Antenna brown except scape and pedicel pale yellow-brown. Legs pale yellow to yellow, except metacoxa, metafemur, apical half of metatibia and tarsus dark brown to black. Wing membranes hyaline, fore wing with distinct dark apical patch, pterostigma brown, veins pale brown to brown.

**Male.** A male specimen from Mount Makiling (Philippines) has a black metafemur and T1 that is almost entirely black, with only a faint red flush along each lateral margin. However, one male from Sabah (Borneo) has a black metafemur and yellow T1, but apical half of T2 is black.

**Host.** Unknown.

**Distribution** (Fig 8)**. Australasian** (Australia-Queensland, Papua New Guinea); **Oriental** (Malaysia [Sabah, Penang], Philippines-Luzon, Singapore); **Palaearctic** (Korea).

**Remarks.** This species is very similar to the Indian species *D. longistria* Gupta & Fernandez, *D. narendrani* Rema & Sheeba, and Chinese *D. chaoi* (Luo & You) in general appearance. Based on our examination, the modified spatulate, upcurved strong setae (Fig 5f) would be useful to distinguish it from the Indian species, and for Chinese *D. chaoi*, the hyaline vein r-m (Figs 5a, 6b), shorter T2 and metacoxa (Fig 5e) may be helpful in diagnosis. *Diolcogaster eclectes* belongs to the *basimacula*–group. One sequence (BIOUG34064-G09, BOLD: ADI0912) from Sumatera Barat, Padang, Indonesia in BOLD is probably this species when compared with the image available on the website. One record of the BIN (BOLD: ADD4731) from Papua New Guinea (Madang) is similar, but with smaller head and obviously enlarged basal flagellomeres.

### *Diolcogaster flavicoxa* Liu & Polaszek, sp. nov.

urn:lsid:zoobank.org:act:978ACC59–6076-49F9-937C-4E65EC417D14

**Material examined.** **HOLOTYPE**: 1♀, BRUNEI, Ulu Temburong National Park (Malaise trap), MC Day, BMNH(E)2011–106, 14.ii–9.iii.1982, No. NHMUK010826524. **PARATYPES**: 17♂♂, same data as holotype except Nos. 010826507, 010826508, 010826509, 010826510, 010826511, 010826512, 010826513, 010826514, 010826515, 010826516, 010826517, 010826518, 010826519, 010826520, 010826521, 010826522, 010826523; 9♂♂, BRUNEI (4°34′N115°7′E, 1800m/3500m), Kuala Belalong FSC (Field Studies Centre) (Malaise), N Mawdsley coll., 24.II.1992/18.V.1991. BMNH(E) 1991–173, Nos. NHMUK010826487, 010826483, 010826492, 010826484, 010826485, 010826491, 010826486, 010826489, 010826493; 1♂, MALAYSIA, DMQI, Danum Plot 6 (Trap MT2). ix/x.2012, No. NHMUK010826490.

**Diagnosis.** Body 2.7 mm long, black, slightly brown, except T1 yellow to pale yellow brown (Fig 1a); head 2.0 × as wide as long; eyes 2.2 × longer than temple in dorsal view; POL:OD:OOL = 1.5:1.1:1.4 (Fig 1b); face with strong rugose punctures, a carina present at upper half midlongitudinally, 0.9 × as wide as high (Fig 1c); antenna with penultimate flagellomere 2.0 × longer than wide (Fig 1d); puncture intervals on mesoscutum hardly one puncture diameter (Fig 1e); propodeum with strong midlongitudinal carina, smooth with few discrete large punctures anterio-laterally (Fig 1h); precoxal sulcus on mesopleuron depressed with sparse punctures, setigerous-punctate mainly underneath its anterior half (Fig 1f); vein r slightly longer than 2-SR, the latter distinctly segmented by r-m; vein r-m reduced to a hyaline point; areolet small, 3-sided; vein 1-CU1 0.8 × 2-CU1 (Fig 1g); metacoxa reaching beyond posterior margin of T4, evenly densely covered with punctures; T1 nearly parallel-sided on dorsal view, 0.6 × longer than basal width, with strong short radiate carinae arising from apical knob of T1; T2 with a median field distinctly broadening towards apex and encircled by wide crenulate grooves, 1.9 × as long as midlength; T3 0.7 × as long as T2 (Fig 1i); ovipositor strong and hook-shaped (Fig 1j).

**Description.** Female. Body length 2.7 mm, fore wing length 2.9 mm (Fig 1a).

***Head.*** 2.0 × as wide as long, 1.1 × as wide as mesoscutum. Eyes 2.2 × longer than temple in dorsal view (Fig 1b). Temple shiny with shallow punctures, constricted behind eyes in dorsal view. Vertex shiny with small sparse punctures, largely polished behind ocelli. Ocelli small, distance between fore and a hind ocellus 0.3 × as long as minor axis of an hind ocellus, POL:OD:OOL = 1.5:1.1:1.4. Frons shiny and polished. Face a little shiny with strong rugose punctures, slightly bulged median longitudinally with a carina at upper half, 0.9 × as wide as high (Fig 1c). Clypeus 2.2 × wider than medial length, rugulose entirely. Tentorial pits of moderate size, distance between tentorial pits 2.5 × as long as distance from pit to eye margin. Length of malar space 2.1 × width of mandible. Antenna 1.3 × longer than body length, with 1st, 2nd and penultimate flagellomeres 3.5, 3.1 and 2.0 × longer than wide, flagellomeres gradually shortened to penultimate flagellomere, closely articulated (Fig 1d).

**Mesosoma.** Length:width:height = 1.6:1.1:1.0. Mesoscutum a little shiny with dense punctures, its intervals hardly one puncture diameter with micro wrinkles, except nearly polished along hind margin; notauli not impressed. Scutellar sulcus wide and straight with 12 carinae inside (Fig 1e). Disc of scutellum a little shiny with small ill-defined punctures entirely, polished at tip, so the posterior, polished band of scutellum is continuous; lateral part of polished band of scutellum reaching hardly to half of disc of scutellum with the anterior triangular part largely polished. Propodeum 2.4 × wider than high, shiny with strong percurrent midlongitudinal carina, smooth with few discrete large punctures anterio-laterally (Fig 1h). Mesopleuron shiny, largely smooth except few punctures on anterior borders, precoxal sulcus depressed with sparse punctures, setigerous-punctate mainly underneath its anterior half (Fig 1f).

***Wings.*** Fore wing (Fig 1g): pterostigma narrow, 2.9 × as long as its widest part; vein 1-R1 1.3 × length of pterostigma; vein r obliquely arising from apical 2/5 of pterostigma, indistinctly longer than maximum width of pterostigma, meeting vein 2-SR at a 138 degree angle, slightly longer than 2-SR, the latter distinctly segmented by r-m; vein r-m reduced to a hyaline point; areolet small, 3-sided; vein m-cu 1.4 × 2-SR + M; vein 1-CU1 0.8 × 2-CU1 and 1.4 × cu-a. Hind wing: narrow, with edge of vannal lobe beyond its widest part very slightly concave and without trace of a fringe of setae, cu-a straight.

***Legs.*** Metacoxa very large, reaching beyond posterior margin of T4, flattened on outer side, evenly densely covered with punctures, the interspaces shiny (Fig 1i). Metafemur 3.3 × as long as its widest part. Metatibia swollen toward apex and 0.8 × as long as metatarsus. Inner metatibial spur much longer than outer one, about 0.8 × as long as metabasitarsus; fourth tarsal segment shorter than fifth tarsal segment (0.9 ×); apical segment of the front tarsus without a spine (Fig 1a). Tarsal claws simple.

***Metasoma*.** Slightly shorter than mesosoma. T1 vertically incurved from lateral view, nearly parallel-sided on dorsal view, 0.6 × longer than basal width, 0.5 × longer than middle width, slightly constricted at apical 1/5, with complete longitudinal groove, strong short radiate carinae arising from apical knob of T1, elsewhere punctate entirely (Fig 1i). T2 trapezoid, with a median field distinctly broadening towards apex and encircled by wide crenulate grooves, elsewhere shiny and polished except crenulate borders, 1.9 × as long as midlength. T3 transverse, 0.7 × as long as T2, membranous, polished, separated from T2 by a crenulate groove. Tergites posterior to T3 more membranous. Setae of metasoma very sparse. Ovipositor sheath narrow, relatively densely setose, without modified setae at apex; ovipositor thick and hook-shaped (Fig 1j). Hypopygium small, evenly sclerotised, smooth with sparse fine setae, not surpassing the last tergite.

***Colour.*** Body black, slightly brown, except T1 yellow to pale yellow-brown (Fig 1a). Palpi white. Tibia purs pale yellow. Antenna brown to dark brown except scape and pedicel pale yellow-brown. Legs yellow, except ventral and tip of metacoxa, tip of metafemur, apical 2/3 of metatibia and most of metatarsus slightly brown. Wing membranes hyaline, slightly fumous, fore wing with pterostigma brown, veins pale brown to brown.

**Male.** Similar to female except antenna longer with penultimate flagellomere 2.5 × longer than wide, T1 longer and hind leg darker with metafemur mostly brown while lateral part of T2 somewhat yellow. Body larger, length 3.5 mm–3.9 mm (Fig 1k).

**Host.** Unknown.

**Distribution** (Fig 8)**. Oriental** (Borneo [Brunei, Malaysia-Sabah]).

**Etymology.** The specific name “*flavicoxa*” derives from the Latin “flavus”(=yellow) and “coxa”, referring to the yellow coxae.

**Remarks.** This species is similar to *S. solitarium* Gupta & Fernández-Triana, but differs in the following: T1 nearly parallel-sided (broadening towards apex in *S. solitarium*); median field on T2 widened backwards (converged backwards in *S. solitarium*); and largely polished on propodeum (strongly densely punctate on propodeum in *S. solitarium*). *Diolcogaster flavicoxa* belongs to the *xanthaspis*–group which is defined by the following combination of characters: i) T1 virtually parallel-sided; ii) T2 with a slightly distinct median field indicated by grooves or, if absent, the median field is smooth and convex, distinguished by longitudinal pits; iii) veins 1-CUa and 1-CUb equal in length; iv) head, scutum, and scutellum rugose-punctate; v) the posterior band of the scutellum sculptured medially; vi) the vannal lobe of the hind wing convex with marginal setae; and vii) the post vertex concave in dorsal view (Nixon 1965). The available image of one BIN (BOLD: ACO6079) including two sequences from Selangor (Malaysia) and four sequences from Padang (Indonesia) in BOLD is similar to *D. flavicoxa* in colour, but with a much longer mesosoma.

### *Diolcogaster hamus* Liu & Polaszek, sp. nov.

urn:lsid:zoobank.org:act:902CCBAD-4155-4E28-9B18-7073674CE58F

**Material examined.** **HOLOTYPE**: 1♀, BRUNEI (4°34′N115°7′E), Kuala Belalong FSC (Field Studies Centre) (Malaise), N Mawdsley coll., v.1991. BMNH(E) 1991–173, No. NHMUK 010826496. **PARATYPE**: same data except No. NHMUK 010826497.

**Diagnosis.** Body 3.3 mm long, black, except T1 yellow (Fig 7a); head 2.1 × as wide as long; eyes 1.3 × longer than temple in dorsal view; POL:OD:OOL = 1.5:1.0:1.2; vertex with coarse punctures between ocelli and eye (Fig 7b); face 1.2 × wider than high (Fig 7c); antenna with penultimate flagellomere 2.0 × longer than wide (Fig 7d); puncture intervals on mesoscutum with micro wrinkles (Fig 7e); propodeum evenly and densely punctate dorsally (Fig 7h); mesopleuron strongly punctate with transverse wrinkles on anterior part (Fig 7i); pterostigma 2.7 × as long as its widest part; vein r 1.4 × 2-SR; areolet medium-sized, 3-sided; vein 1-CU1 0.6 × 2-CU1 (Fig 7f); metacoxa reaching nearly apex of metasoma (Fig 7i); T1 gradually widened towards apex, 1.5 × longer than basal width (Fig 7g); T2 with a median field parallel-sided and encircled by narrow crenulate grooves, elsewhere with strong striations and ill-defined punctures in between, 1.8 × as long as midlength; T3 slightly longer than T2, with median polished field roundly narrowed towards apex, sparsely striate laterally; ovipositor thick basally and distinctly attenuated apically (Fig 7i).

**Description.** Female. Body length 3.3 mm, fore wing length 3.3 mm (Fig 7a).

***Head.*** 2.1 × as wide as long, nearly as wide as mesoscutum. Eyes 1.3 × longer than temple in dorsal view (Fig 7b). Temple a little shiny with shallow punctures, constricted behind eyes in dorsal view. Vertex shiny with coarse punctures between ocelli and eye. Ocelli medium, distance between fore and a hind ocellus 0.4 × as long as minor axis of an hind ocellus, POL:OD:OOL = 1.5:1.0:1.2. Frons shiny and polished. Face less shiny with setigerous-punctate, weakly rugulose below sockets, a carina present at upper half midlongitudally, 1.2 × wider than high (Fig 7c). Clypeus 2.4 × wider than medial length, nearly smooth entirely. Tentorial pits of moderate size, distance between tentorial pits 3.7 × as long as distance from pit to eye margin. Length of malar space 0.9 × width of mandible. Antenna 1.2 × longer than body length, with 1st, 2nd and penultimate flagellomeres 2.9, 2.7 and 2.0 × longer than wide, flagellomeres gradually shortened to penultimate flagellomere, closely articulated (Fig 7d).

***Mesosoma*.** Length:width:height = 1.5:1.0:1.3. Mesoscutum a little shiny with dense punctures, its intervals less than one puncture diameter and with micro wrinkles, narrowly polished along hind margin; notauli not impressed. Scutellar sulcus wide and straight with 10 carinae inside (Fig 7e). Disc of scutellum a littly shiny with similar punctures as mesoscutum, polished at tip, so the posterior, polished band of scutellum is continuous; lateral part of polished band of scutellum reaching over half of disc of scutellum with the anterior triangular part narrowly depressed with shallow sparse punctures. Propodeum 3.1 × wider than high, shiny with strong percurrent midlongitudinal carina, evenly and densely punctate dorsally, spiracles large, enclosed by costulae (Fig 7h). Mesopleuron shiny, strongly punctate with transverse wrinkles on anterior part, weekly rugulose punctate on lower half, polished on medio-posterior part with few discrete punctures underneath, precoxal sulcus indistinct covered by weak rugosity (Fig 7i).

***Wings.*** Fore wing (Fig 7f): pterostigma large, 2.7 × as long as its widest part; vein 1-R1 1.2 × length of pterostigma; vein r slightly obliquely arising from apical 2/5 of pterostigma, 1.9 × longer than maximum width of pterostigma, meeting vein 2-SR at a 129 degree angle, 1.4 × 2-SR; vein r-m 0.5 × 2-SR; areolet medium, 3-sided; vein m-cu 1.5 × 2-SR + M; vein 1-CU1 0.6 × 2-CU1 and as long as cu-a. Hind wing: narrow, with edge of vannal lobe beyond its widest part nearly straight and with minuate setae; cu-a straight.

***Legs.*** Metacoxa large, reaching nearly apex of metasoma, flattened on outer side, evenly covered with sparse minute punctures, which coarser anterio-dorsally (Fig 7i). Metafemur 3.5 × as long as its widest part. Metatibia gradually swollen toward apex and 0.9 × as long as metatarsus. Inner metatibial spur much longer than outer one, 0.7 × as long as metabasitarsus; fourth tarsal segment as long as fifth tarsal segment; apical segment of the front tarsus without a spine. Tarsal claws simple.

***Metasoma*.** About as long as mesosoma. T1 mediumly incurved from lateral view, gradually widened towards apex, 1.5 × longer than basal width, with complete longitudinal groove attaching to weak short radiate carinae arising from apical knob of T1, elsewhere densely punctate entirely (Fig 7g). T2 almost rectangular, with a median field parallel-sided and encircled by narrow crenulate grooves, elsewhere with strong striations and ill-defined punctures in between, 1.8 × as long as midlength. T3 transverse, slightly longer than T2, with median polished field roundly narrowed towards apex, sparsely striate laterally. Tergites posterior to T3 contracted, membranous. Setae of metasoma very sparse. Ovipositor sheath narrow, with few setae apically, without modified setae; ovipositor thick basally and distinctly attenuated apically. Hypopygium not large, evenly sclerotised, smooth with small punctures, not surpassing the last tergite (Fig 7i).

***Colour.*** Body black, except T1 yellow (Fig 7a). Palpi and tibia spurs pale yellow. Antenna brown to dark brown except scape, pedicel and basal two to three flagellomeres pale yellow-brown. Legs pale yellow to yellow, except mid femur, metacoxa, metafemur, apical half of metatibia and metatarsus dark brown to black. Wing membranes hyaline, fore wing marked with brown patches at apex, pterostigma brown, veins pale brown to brown, r-m hyaline.

**Variation.** Body length 3.2–3.3 mm, fore wing length 3.3–3.4 mm

**Male.** Unknown.

**Host.** Unknown.

**Distribution** (Fig 8)**. Oriental** (Borneo [Brunei]).

**Etymology.** The specific name “*hamus*”, derives from the Latin, referring to the hook-like shape of ovipositor.

**Remarks.** This species is similar to *D. dolichogaster* Liu & Polaszek, sp. nov., but differs in the following: vertex shiny with coarse punctures between ocelli and eye (nearly polished in *D. dolichogaster*); propodeum evenly and densely punctate dorsally (largely polished in *D. dolichogaster*); and face 1.2 × wider than high (narrower in *D. dolichogaster*). *Diolcogaster hamus* belongs to the *basimacula*–group. The available images of one BIN (BOLD: ACO7103) from Selangor (Malaysia) and one BIN (BOLD: AAH1210) from Padang (Indonesia) are similar to this new species in colour except with paler antennae, and the hind coxa extends to only two thirds of metasoma while in *D*. *hamus* it reaches the apex of the metasoma.

### *Diolcogaster parallela* Liu & Polaszek, sp. nov.

urn:lsid:zoobank.org:act:65BCE4BE-4412-4B89-A122-35A89ECDC12B

**Material examined** (NHMUK)**. HOLOTYPE**: 1♀, BRUNEI, Ulu Temburong N.P. (Malaise trap), MC Day, BMNH(E)2011−106, 14.ii–9.iii.1982, No. NHMUK010826506. **PARATYPE**: 1♀, same data as holotype except No. 010826504; 1♀, MALAYSIA, Borneo, Sarawak, 4^th^ Div. Gn. Mulu. RGS Exp. (Malaise trap), NM Collins, v.1978, No. NHMUK010826505.

**Diagnosis.** Body 3.7 mm long, black, slightly brown (Fig 2a); head 1.8 × as wide as long; eyes 2.2 × longer than temple in dorsal view; POL:OD:OOL = 1.3:1.0:1.3; vertex with denser punctures behind ocell (Fig 2b); face nearly as wide as high, with more defined punctures medially, setigerous-punctate laterally (Fig 2c); antenna with penultimate flagellomere 2.8 × longer than wide (Fig 2d); puncture intervals on mesoscutum polished and hardly one puncture diameter (Fig 2e); propodeum with areolate-rugose sculptures medially, smooth elsewhere (Fig 2i); mesopleuron smooth on basal and apical areas except dense punctures on anterior borders (Fig 2f); vein r 1.7 × longer than maximum width of pterostigma; vein 1-CU1 0.6 × 2-CU1 and 0.9 × cu-a (Fig 2g); hind wing with edge of vannal lobe beyond its widest part nearly straight and with minute setae; metacoxa reaching close to posterior margin of T4 (Fig 2f); T1 nearly parallel-sided, 0.8 × longer than basal width, as long as middle width (Fig 2i); T2 with a median field parallel-sided and encircled by crenulate grooves, elsewhere with strong striations and ill-defined punctures in between, 1.6 × as long as midlength; T3 0.9 × as long as T2, with similar sculptures to T2 on anterior half, nearly polished along posterior margin; hypopygium distinctly surpassing the last tergite(Fig 2f).

**Description.** Female. Body length 3.7 mm, fore wing length 3.8 mm (Fig 2a).

***Head.*** 1.8 × as wide as long, nearly as wide as mesoscutum. Eyes 2.2 × longer than temple in dorsal view (Fig 2b). Temple a little shiny with shallow punctures, constricted behind eyes in dorsal view. Vertex shiny with small sparse punctures which more denser behind ocelli. Ocelli small, distance between fore and a hind ocellus 0.4 × as long as minor axis of an hind ocellus, POL:OD:OOL = 1.3:1.0:1.3. Frons shiny and polished. Face shiny with more defined punctures medially, setigerous-punctate laterally, weakly rugulose below sockets, a carina present at upper half midlongitudally, nearly as wide as high (Fig 2c). Clypeus 3.1 × wider than medial length, shallowly and sparsely punctate entirely. Tentorial pits of moderate size, distance between tentorial pits 3.5 × as long as distance from pit to eye margin. Length of malar space 1.2 × width of mandible. Antenna 1.2 × longer than body length, with 1st, 2nd and penultimate flagellomeres 3.3, 3.1 and 2.8 × longer than wide, flagellomeres gradually shortened to penultimate flagellomere, closely articulated (Fig 2d).

***Mesosoma*.** Length:width:height = 1.6:1.0:1.3. Mesoscutum a little shiny with dense punctures, with its intervals less than one puncture diameter but no micro wrinkles, polished along hind margin; notauli not impressed. Scutellar sulcus wide and straight with 10 carinae inside (Fig 2e). Disc of scutellum slightly shiny with small punctures, polished at tip, so the posterior, polished band of scutellum is continuous; lateral part of polished band of scutellum reaching hardly to half of disc of scutellum with the anterior triangular part scattered with small punctures. Propodeum 2.3 × wider than high, shiny with strong percurrent midlongitudinal carina, dorsal part with areolate-rugose sculptures medially, smooth elsewhere, spiracles large, enclosed by costulae (Fig 2i). Mesopleuron shiny, smooth on basal and apical areas except dense punctures on anterior borders, precoxal sulcus depressed with small punctures (Fig 2f).

***Wings.*** Fore wing (Fig 2g): pterostigma narrow, 3.5 × as long as its widest part; vein 1-R1 1.3 × length of pterostigma; vein r strongly obliquely arising from apical 2/5 of pterostigma, 1.7 × longer than maximum width of pterostigma, meeting vein 2-SR at a 91 degree angle, 1.6 × 2-SR; vein r-m reduced to a hyaline point; areolet small, 3-sided; vein m-cu 1.3 × 2-SR + M; vein 1-CU1 0.6 × 2-CU1 and 0.9 × cu-a. Hind wing (Fig 2h): narrow, with edge of vannal lobe beyond its widest part nearly straight and with minute setae; cu-a nearly straight.

***Legs.*** Metacoxa large, reaching close to posterior margin of T4, flattened on outer side, evenly densely covered with small punctures, the interspaces shiny (Fig 2f). Metafemur 3.6 × as long as its widest part. Metatibia swollen toward apex and 0.8 × as long as metatarsus. Inner metatibial spur much longer than outer one, 0.7 × as long as metabasitarsus; fourth tarsal segment as long as fifth tarsal segment; apical segment of the front tarsus without a spine (Fig 2a). Tarsal claws simple.

***Metasoma*.** Slightly (0.9 ×) shorter than mesosoma. T1 mediumly incurved from lateral view, nearly parallel-sided on dorsal view, 0.8 × longer than basal width, as long as middle width, with complete longitudinal groove attaching to strong short radiate carinae arising from apical knob of T1, elsewhere deeply punctate entirely (Fig 2i). T2 trapezoid, with a median field parallel-sided and encircled by crenulate grooves, elsewhere with strong striations and ill-defined punctures in between, 1.6 × as long as midlength. T3 transverse, 0.9 × as long as T2, membranous on posterior margin, with similar sculptures to T2 on anterior half, nearly polished along posterior margin. Tergites posterior to T3 more membranous. Setae of metasoma very sparse. Ovipositor sheath narrow, relatively densely setose, without modified setae at apex; ovipositor thin and slightly curved (Fig 2f). Hypopygium large, evenly sclerotised, smooth with small punctures, distinctly surpassing the last tergite.

***Colour.*** Body black, slightly brown on head and metasoma (Fig 2a). Palpi and tibia spurs pale yellow. Antenna brown to dark brown except scape and pedicel pale yellow-brown. Legs yellow, except most of mid femur and tibia, most of metacoxa, femur, apical 2/3 of tibia and most of metatarsus brown. Wing membranes hyaline, distinctly infumate, fore wing with pterostigma brown, veins pale brown to brown.

**Variation.** No distinct differences observed among specimens. Body length 3.6–7 mm, fore wing length 3.7–3.8 mm.

**Male.** Unknown.

**Host.** Unknown.

**Distribution** (Fig 8)**. Oriental** (Borneo [Brunei, Malaysia-Sarawak]).

**Etymology.** The specific name “*parallela*” derives from the Greek and Latin “parallelos”, referring to its parallel-sided median field on T2.

**Remarks.** This species is similar to *D. flavicoxa* Liu & Polaszek, sp. nov., but can be differentiated by characters in the key above. When compared with other Oriental species, it resembles *D. punctata*, but differs in the following: vertex with small punctures (strongly rugose in *D. punctata*); vein r on fore wing 1.7 × longer than maximum width of pterostigma (0.7 × in *D. punctata*); and deeply punctate entirely on T1 (apical half of T1 smooth in *D. punctata*). *Diolcogaster parallela* belongs to the *xanthaspis*–group. We examined the images of BINs deposited in BOLD from surrounding countries without finding a match.

### *Diolcogaster urios* (Nixon).

*Protomicroplitis urios*: Nixon 1965: 243 [6]; Rao & Chalikwar 1970: 113 [20].

*Diolcogaster urios*: Mason 1981: 115 [3]; Fernández-Triana 2015: 534 [11]; Fernández-Triana et al. 2020: 429 [2].

**Material examined. HOLOTYPE**: 1♀, MALAYSIA, Borneo, Sabah, Sandakan, CF Baker coll., type No. USNMENT01932083.

**Diagnosis.** Head red-brown (Fig 3e); disc of scutellum with similar dense punctuation as mesoscutum (Fig 3f); posterior, polished band of scutellum interrupted at middle by a small area of rugosity; vein r of fore wing very long, meeting r-m at the junction of 3-SR (Fig 3b); temple very short, strongly constricted behind eyes, eyes 3.4 × longer than temple in dorsal view (Fig 3e); OOL relatively short, 0.8 × of OD; face red-brown and strongly rugose-punctate on sides (Fig 3c); mesosoma almost black (Figs 3a, 3f–3g); metacoxa red-yellow (Fig 3a); metatibia about as long as metatarsus (Fig 3a).

**Redescription.** Female. Body length 2.9 mm, fore wing length 3.0 mm (Fig 3a)

***Head.*** 2.3 × as wide as long, nearly as wide as mesoscutum. Eyes 3.4 × longer than temple in dorsal view (Fig 3e). Temple shiny with shallow punctures, strongly constricted behind eyes in dorsal view. Vertex shiny with ill-defined, small punctures between ocelli and eye, highly polished behind ocelli. Ocelli small, distance between fore and a hind ocellus 0.4 × as long as minor axis of an hind ocellus, POL:OD:OOL = 1.6:1.3:1.0. Frons shiny and polished. Face a little shiny with strong rugose punctures on sides, distinctly bulged median longitudinally with a carina at upper half, about as wide as high. Clypeus 4.0 × wider than medial length, weakly rugulose. Tentorial pits of moderate size, distance between tentorial pits 2.5 × as long as distance from pit to eye margin. Length of malar space 1.2 × width of mandible (Figs 3c, 3d). Antenna much longer than body length, with 1st and 2nd flagellomeres 3.5 and 3.3 × longer than wide, flagellomeres gradually shortened to penultimate flagellomere, closely articulated, four apical flagellomeres missing (Fig 3i).

**Mesosoma.** Length:width:height = 1.3:1.3:1.0. Mesoscutum a little shiny with dense punctures, its intervals less than half puncture diameter with micro punctuation, except narrowly polished along hind margin; notauli not impressed (Fig 3f). Scutellar sulcus wide and straight with 10 carinae inside. Disc of scutellum slightly shiny with similar punctuation as mesoscutum, posterior, polished band of scutellum interrupted at middle by a small area of rugosity. Propodeum with strong percurrent midlongitudinal carina and coarse punctures laterally. Mesopleuron shiny, largely smooth except few punctures on anterior borders, precoxal sulcus depressed with sparse punctures.

***Wings.*** Fore wing (Fig 3b): pterostigma medium- sized, 2.8 × as long as its widest part; vein 1-R1 1.7 × length of pterostigma; vein r slightly obliquely arising from apical 2/5 of pterostigma, 1.5 × longer than maximum width of pterostigma, meeting vein 2-SR at a 121 degree angle, 2.4 × longer than 2-SR; vein r-m connected to the meeting point of r and 2-SR; areolet large, 3-sided; vein m-cu as long as 2-SR + M; vein 1-CU1 nearly 0.9 × 2-CU1 and 1.4 × cu-a. Hind wing: narrow, with edge of vannal lobe beyond its widest part very slightly concave and without trace of a fringe of setae, cu-a straight.

***Legs.*** Metacoxa large, reaching beyond posterior margin of T3, flattened on outer side, evenly sparsely covered with small punctures, the interspaces shiny. Metafemur 3.5 × as long as its widest part (Fig 3g). Metatibia swollen toward apex and about as long as metatarsus. Inner metatibial spur much longer than outer one, about 0.8 × as long as metabasitarsus; fourth tarsal segment indistinctly longer than fifth tarsal segment; apical segment of the front tarsus without a spine (Fig 3h). Tarsal claws simple.

***Metasoma*.** Nearly 1.2 × longer than mesosoma. T1 slightly incurved from lateral view, narrow, parallel-sided, about 1.5 × as long as its middle width, with complete longitudinal groove. T2 with a slightly distinct median field, not triangularly widened posteriorly. T3 smooth, polished and never forming with T2 a carapace. Tergites posterior to T3 more membranous. Setae of metasoma very sparse. Ovipositor sheath narrow, relatively sparsely setose, without modified setae at apex. Hypopygium medium-sized, evenly sclerotised, smooth with sparse fine setae, not surpassing the last tergite (Fig 3h).

***Colour.*** Body black, slightly brown, except head red-brown and metasoma red-yellow (excluding tergites posterior to T1) (Fig 3a). Palpi pale yellow. Tibia spurs red-yellow. Antenna red-brown to brown except scape and pedicel slightly yellow-brown. Legs red-yellow, except slightly red-brown on apical third of metatibia. Wing membranes hyaline, fore wing with pterostigma brown, veins pale brown to brown.

**Male.** Unknown.

**Host.** Unknown.

**Distribution** (Fig 8)**. Oriental** (Borneo (Malaysia**-**Sabah).

**Remarks.** This species is very similar to *D. mellea* Nixon, but can be differentiated by the following: mesoscutum black (yellow with faint infuscate spot at shoulder in *D. mellea*); vein r much longer, 2.4 × longer than 2-SR (1.4 × in *D. mellea*); fore wing with areolet, vein r-m more distinct (without areolet for vein r-m not indicated in *D. mellea*). *Diolcogaster urios* belongs to the *xanthaspis*–group. We examined the images of BINs deposited in BOLD from surrounding countries without finding a match.

## Discussion and conclusion

The genus *Diolcogaster* is readily distinguishable from other genera within Microgastrinae despite its morphologically diverse species composition. However, its diagnostic key characters do not appear to be uniquely derived. As a result, *Diolcogaster* is divided into multiple distinct species–groups, some of which seem to share closer relationships with other genera (e.g., [9]). Two main species–groups are found and well distinguished in this study: the *basimacula*–group (*D. dolichogaster*, *D. eclectes*, and *D. hamus*) and the *xanthaspis*–group (*D. flavicoxa*, *D. parallela* and *D. urios*). The *basimacula*–group is probably the largest species group within *Diolcogaster* with several New and Old World species. This group is rendered demonstrably paraphyletic by the genus *Buluka*, which differs primarily in having T1–T3 fused into a complete carapace, and the antenna does not have the special groove and sensilla [16]. However, the *xanthaspis*–group is a relatively small species group comprising taxa from the Oriental, Australasian, and New World regions.

Some species–groups within *Diolcogaster* are likely not monophyletic, owing to a high degree of homoplasy in morphological characters [9,16]. As a result, a thorough revision of the species–groups and associated species within *Diolcogaster* is urgently needed, as some taxa probably belong to other genera and are not true members of *Diolcogaster*. For instance, several species from the Afrotropical, Australasian, and Oriental regions appear to belong to different genera. Notably, the *euterpus*–group, which is endemic to the Australasian region, may be elevated to generic status in future studies due to its highly distinctive morphology, particularly its completely smooth body and pectinate tarsal claws [6,9]. The development of a natural classification for the genus will require a comprehensive phylogenetic and phylogenomic framework, incorporating broader taxon sampling across the tribe Cotesiini (*sensu* [3]).

**Fig 9:**
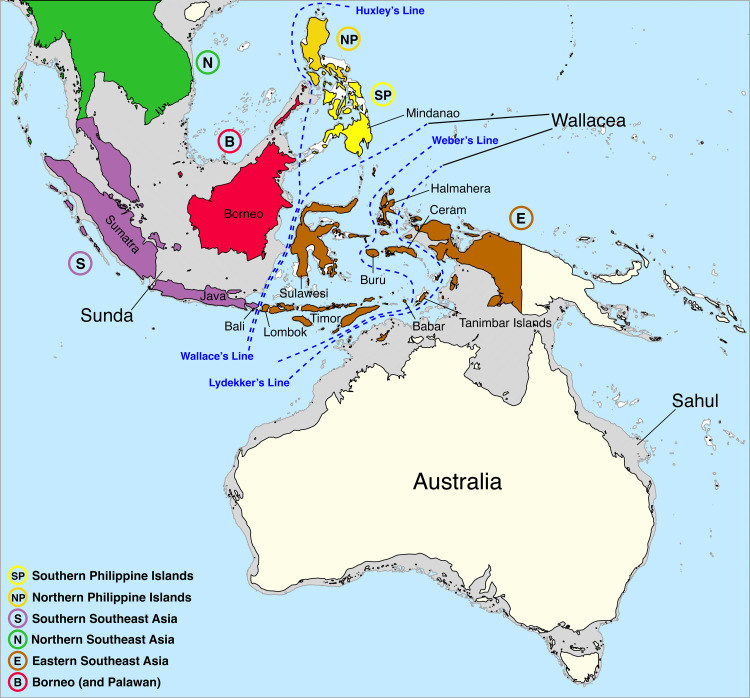
Biogeographical map of Southeast Asia showing the main regional divisions (Sunda, Wallacea, and Sahul), major biogeographic demarcation lines (e.g., Wallace’s, Weber’s, Huxley’s, and Lydekker’s lines), and the position of Borneo within the Borneo (B) biogeographic zone. Coloured regions represent distinct biogeographic subregions relevant to faunal distribution patterns. Source: from ©Wikipedia, modified and reproduced under a CC BY license, with permission from Wikipedia [2025].

The geological history of Southeast (SE) Asia and the Southwest Pacific is highly complex [23]). Sundaland is a biogeographical region of SE Asia that corresponds to a larger landmass periodically exposed over the past 2.6 million years during periods of lower sea levels [24–25]. It includes Bali, Borneo, Java, Sumatra, and their surrounding small islands, as well as the Malay Peninsula on the Asian mainland (Fig 9). In this work, *D. dolichogaster* and *D. eclectes*is were found in the Malay Peninsula as well, apart from Borneo, demonstrating the connection between these two areas, both as parts of Sundaland. *D. eclectes*, however, has a wider expansion, ranging from Palaearctic, Oriental to Australasian. Others seems more endemic to tropical (all located in Borneo only for now). Historically, only *D. eclectes* was recorded from Peninsular Malaysia and three (with two species of *basimacula*–group and one aberrant Wallacea species *D. euterpe*) from Indonesia, showing the ubiqutous distribution of the *basimacula*–group in Sundaland, and species differences between two areas separated by the two major biogeographical boundaries, Huxley’s Line and Wallace’s Line (Fig 9). Compared to the seven species–groups, along with six unplaced species found from Australasia by Saeed et al [9], and dozens of BINS in nearby countries, Borneo, in particular, remains relatively untouched, highlighting the need for further examination of specimens from this region, especially through molecular studies and increased sampling from these habitats. We acknowledge the failure in extracting DNA from old materials in the collection for this prelimary study of this area and trying to get fresh material in the near future which is important for species delimitation, especially the undescribed BINs in BOLD. The species and genera of this island, particularly within the subfamily Microgastrinae [26] and other parasitoid wasp groups [27–28], appear to be unique and show their potential in future biocontrol implemention, making focused research in this area essential.
